# Retraining and control therapy: sense of control and catastrophic symptom expectations as targets of a cognitive behavioral treatment for pediatric functional seizures

**DOI:** 10.3389/fpsyt.2025.1610446

**Published:** 2026-02-12

**Authors:** Caroline Watson, Badhma Valaiyapathi, Jerzy P. Szaflarski, Burel R. Goodin, Aaron D. Fobian

**Affiliations:** 1 Department of Psychology, University of Alabama at Birmingham, Birmingham, AL, United States; 2 Department of Psychiatry and Behavioral Neurobiology, University of Alabama at Birmingham, Birmingham, AL, United States; 3 Department of Neurology, University of Alabama at Birmingham, Birmingham, AL, United States; 4 Department of Anesthesiology, Washington University in St. Louis, St. Louis, MO, United States

**Keywords:** functional neurological disorder, functional seizures, psychogenic non-epileptic seizures (PNES), randomized controlled trial, retraining and control therapy (ReACT)

## Abstract

This manuscript outlines the methodology of a randomized controlled trial (Clinical Registry #: NCT06007053) that aims to assess sense of control and catastrophic symptom expectations as targets of Retraining and Control Therapy (ReACT) a cognitive behavioral treatment for pediatric functional seizures (FS). Participants will complete baseline assessments of treatment targets and other outcome measures. The intervention will consist of either 12 sessions of ReACT or supportive therapy (i.e., unstructured, discussion-oriented therapy). Treatment targets and other outcome measures will be assessed again at one-week post-treatment and two-months post-treatment. The findings of the outlined study will contribute to the growing body of research on FS treatment by evaluating the effectiveness of ReACT in modifying key treatment targets and improving patient outcomes. If successful, ReACT may offer a structured, evidence-based approach for addressing FS in pediatric populations. Future directions include further refinement of the intervention and exploration of factors that may contribute to symptom persistence and remission.

**Clinical Trial Registration:**

https://clinicaltrials.gov/study/NCT05096273, identifier NCT05096273.

## 1 Introduction

Functional seizures (FS) are a type of functional neurological disorder (FND; previously known as conversion disorder) characterized by seizure-like symptoms without associated epileptiform activity in the brain (1). In seizure clinics, approximately 20% of the 70 million patients evaluated for seizures each year are diagnosed with FS, making it a significant clinical and societal burden (2). FS typically start in adolescence or early adulthood (3) and are severely debilitating to patients and their families (4). For example, children and adolescents with FS are at increased risk of academic difficulties due to bullying and absenteeism (5) and consistently report poor peer relationships and withdrawal from extracurricular and social activities (6). With regard to parents and families of children and adolescents with FS, absenteeism from work is common and associated with significant financial burden (7, 8).

FND care providers often explain FS as the result of stress, psychopathology, or trauma (9, 10). However, previous studies assessing the use of the antidepressant sertraline to treat FS have found no significant difference with regard to FS frequency when compared to a placebo (11, 12). While rates of psychiatric comorbidities are higher among children and adolescents with FS as compared with healthy controls, childhood abuse has not been identified as a risk factor for pediatric FS, and psychiatric comorbidities have been found to be similar between adolescents with FS and controls matched on age, sex, race and household income (13). Therefore, novel FS treatment targets beyond mood and trauma are needed (14).

Recent research has revealed two potential targets for treating FS. First, children and adolescents with FS have been found to experience greater catastrophic symptom expectations (i.e., the interpretation of physical sensations as injurious, intense and disturbing) as compared with their siblings (15) and peers with epilepsy (16). Second, previous literature has demonstrated impaired sense of control (i.e., the extent to which a person perceives or feels in control) in children and adolescents with FS (17). Theoretical frameworks support these targets, including predictive coding and Bayesian models which suggest that functional symptoms may arise from inaccurate interpretations of bodily signals and an imbalance between sensory input and prior expectations (18–20). Neuroimaging studies demonstrate disruptions in neural networks involved in sense of agency and sensorimotor integration (21, 22). These perspectives strengthen the rationale for targeting catastrophic symptom expectations and sense of control in interventions. Retraining and Control Therapy (ReACT)—the only treatment for pediatric FS supported by outcomes of a pilot randomized controlled trial (23)—aims to treat pediatric FS by addressing these targets. Specifically, ReACT is a cognitive behavioral therapy (CBT)-based treatment which aims to 1) change catastrophic symptom expectations related to involuntary FS and 2) increase sense of control through habit reversal, a well-established treatment for involuntary tics (24). Compared to supportive therapy, ReACT resulted in significantly reduced FS frequency with 82% of children remaining FS-free for ≥2 months (23). This is promising given the lack of a gold standard treatment for pediatric FS. Confirming these factors as treatment targets will be critical in advancing the development of an evidence-based treatment for FS. Further, a preliminary study suggests sense of control improves after ReACT (25).

The present study aims to compare post-treatment differences in sense of control and catastrophic symptom expectations (treatment targets) and between the ReACT and supportive therapy. Secondary study aims include assessing whether changes in sense of control and catastrophic symptom expectations are associated with change in FS frequency and whether there is an interaction between the two treatment targets.

## 2 Methods and analysis

### 2.1 Study design

The study is a single site two-arm parallel randomized controlled trial for adolescents with FS. Participants will undergo 12 sessions of either ReACT or supportive therapy. The study will be conducted at the University of Alabama at Birmingham (UAB) and will include 80 participants with FS and their family member enrolled from March 2024 to December 2026. The study was approved by the Institutional Review Board (IRB) at UAB and is registered at ClinicalTrials.gov under registration number #NCT06007053.

### 2.2 Selection of subjects

Inclusion criteria will include ages 11- to 18-year-olds with a diagnosis of FS as confirmed by video electroencephalogram (VEEG). Self- and/or parent-reported exclusion criteria will include comorbid epilepsy, less than 4 FS per month, other paroxysmal non-epileptic events, severe intellectual disability, participation in other therapy, and severe mental illness characterized by active psychosis. Other exclusion criteria will be assessed by research assistants and will include blood pressure >130/80 mmHg for adolescents 13 or older and either systolic or diastolic blood pressure greater than or equal to 95% based on sex and age for children under 13. Because one of the study’s primary outcome tasks, the Cold Pressor Test (CPT), is contraindicated for individuals with elevated blood pressure, these criteria are in place to minimize risk for participants who may have hypertension.

### 2.3 Study procedures and interventional methods

Adolescents who are referred to the PI and last author’s (AF) FND Treatment Clinic at UAB will be informed of the study, and if interested, asked to confirm eligibility as a means of recruitment. The Epilepsy Monitoring Unit (EMU) at Children’s of Alabama will also contribute to recruitment such that patients diagnosed with FS will be informed of the study, and if interested, EMU staff will call study staff who will go to the EMU and provide additional study information. Given 11 of the 12 therapy sessions were approved to be held via telehealth, this will allow for the enrollment of a regionally eclectic sample of participants.

**Figure 1:**
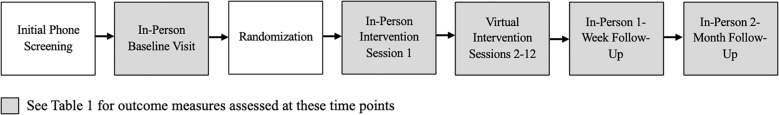
Study design from recruitment to follow-up.

Adolescents who meet inclusion criteria during an initial screening call and are interested in participating will be invited to attend an in-person baseline lab visit (**Figure 1**). Upon arrival, a research assistant will confirm the number of FS they experienced in the last month. If they report at least 4 FS in the last month, eligibility will be confirmed. The research assistant will then discuss study details with the parent and participant, including the purpose, procedures, and risks and benefits of participation the study. The parent/guardian will sign informed consent, and participants will sign an informed consent and/or assent. In accordance with Institutional Review Board protocol at the University of Alabama at Birmingham, a HIPPA authorization form will also be reviewed and signed at the same time as the consent. Immediately after signing all appropriate forms, participants and their parents will complete the baseline in-person lab visit. This visit includes parent and adolescent questionnaires, the CPT, the Magic and Turbulence Task, and cortisol samples.

Following their baseline appointment, participants will be randomized to complete either 12 weekly sessions of ReACT or supportive therapy (see below for detailed description) and complete questionnaires before each therapy session. After treatment is completed, participants will complete follow-up visits consistent with the baseline visit at 1-week and 2-months after treatment.

#### 2.3.1 Randomization and blinding

Randomization to either ReACT or supportive therapy will be prepared in a closed envelope fashion and prepared by PROC PLAN in SAS version 9.4, and a unique randomization number will be generated for each participant. The randomization will be performed in random block sizes ranging from 2 to 4. Researchers who conduct baseline and follow-up assessments will be blinded to which intervention the patient was assigned. The statistician and data manger will also be blinded to which intervention the patient was assigned when analyzing data. Unblinding will take place at the conclusion of the study, following the completion of data analysis.

### 2.4 Primary outcome measures

Primary outcomes (i.e., sense of control and catastrophic symptom expectations) will be assessed by the Magic and Turbulence Task, and salivary cortisol response, pain catastrophizing questionnaire, and pain tolerance to the CPT. All primary outcome measures will be assessed at baseline and one week and two months after the conclusion of treatment by research assistants (**Table 1**). Importantly, these tasks are intended to serve as representative measures of broader biobehavioral processes than as exhaustive representations of their respective constructs, which likely manifest across contexts.

**Table 1:** Outcome measures.

Outcome Measures	Baseline Visit	Session 1	Weekly	1-Week Follow-up	2-Month Follow-up
Primary outcomes					
Magic and Turbulence Task	X			X	X
Childhood Anxiety Sensitivity Index	X			X	X
Pain Catastrophizing Scale for Children	X			X	X
Salivary Cortisol Response	X			X	X
Pain Sensitivity (Cold Pressor Test)	X			X	X
Perceived Pain Severity (Cold Pressor Test)	X			X	X
Secondary outcomes					
FND and somatic symptoms	Baseline Visit	Session 1	Weekly	1-Week Follow-up	2-Month Follow-up
Functional Seizure Diaries	X	X	X	X	X
Tic Questionnaire (Parent/Child; If Reported)	X			X	X
Other FND Symptoms Questionnaire	X			X	X
The Children’s Somatic Symptom Inventory-24	X			X	X
LEVEL-2 Somatic Symptom	X			X	X
Mood and quality of life	Baseline Visit	Session 1	Weekly	1-Week Follow-up	2-Month Follow-up
Anxiety Sensitivity Index	X			X	X
The Revised Children’s Anxiety and Depression Scale	X			X	X
Dysfunctional Attitude Scale (DAS-9)	X				
Quality of Life in Epilepsy for Adolescents	X			X	X
Functional Disability Index	X			X	X
Single Quality of Life Question	X	X	X	X	X
Rosenberg Self-Esteem Scale		X			
The Columbia Suicide Severity Rating Scale	X			X	X
Family dynamics and functioning	Baseline Visit	Session 1	Weekly	1-Week Follow-up	2-Month Follow-up
The Impact on Family Scale	X			X	X
Parenting Style: PSDQ Short Version (Parent/Spouse)		X		X	
Beaver’s Self-Report Family Inventory (Child)	X				
Beaver’s Self-Report Family Inventory (Parent)		X		X	X
Treatment engagement, beliefs, and alliance	Baseline Visit	Session 1	Weekly	1-Week Follow-up	2-Month Follow-up
Treatment Preference Questionnaire	X				
Credibility/Expectancy Questionnaire (Parent/Child)		X		X	X
Revised Helping Alliance Questionnaire (Parent/Child)		X			
Revised Helping Alliance Questionnaire- II (Therapist)			X		
Clinical Global Impressions Index (Therapist)		X	X		
Child Weekly Therapy Questionnaire		X	X		
Parent Weekly Therapy Questionnaire		X	X		
Child Follow-up Questionnaire				X	X
Parent Follow-up Questionnaire				X	X
Illness cognitions and sense of control					
Single Sense of Agency Question	X			X	X
FND Locus of Control: MHLC Scales, Form C	X			X	X
FND Locus of Control: MHLC Scales, Form A		X		X	X
Mishel Uncertainty in Illness Questionnaire		X			
Sick Role: Illness Cognitions Scale		X			
Cognitive and sociodemographic background					
Demographics Questionnaire	X				
Shipley-2 IQ Screener	X				
Child Mini Form	X				
Parent Mini Form	X				
Healthcare Related Stigma Questionnaire (Parent/Child)	X				
The Childhood Trauma Questionnaire		X			
COVID-19 FND Questionnaire		X			

#### 2.4.1 Sense of control

**Figure 2:**
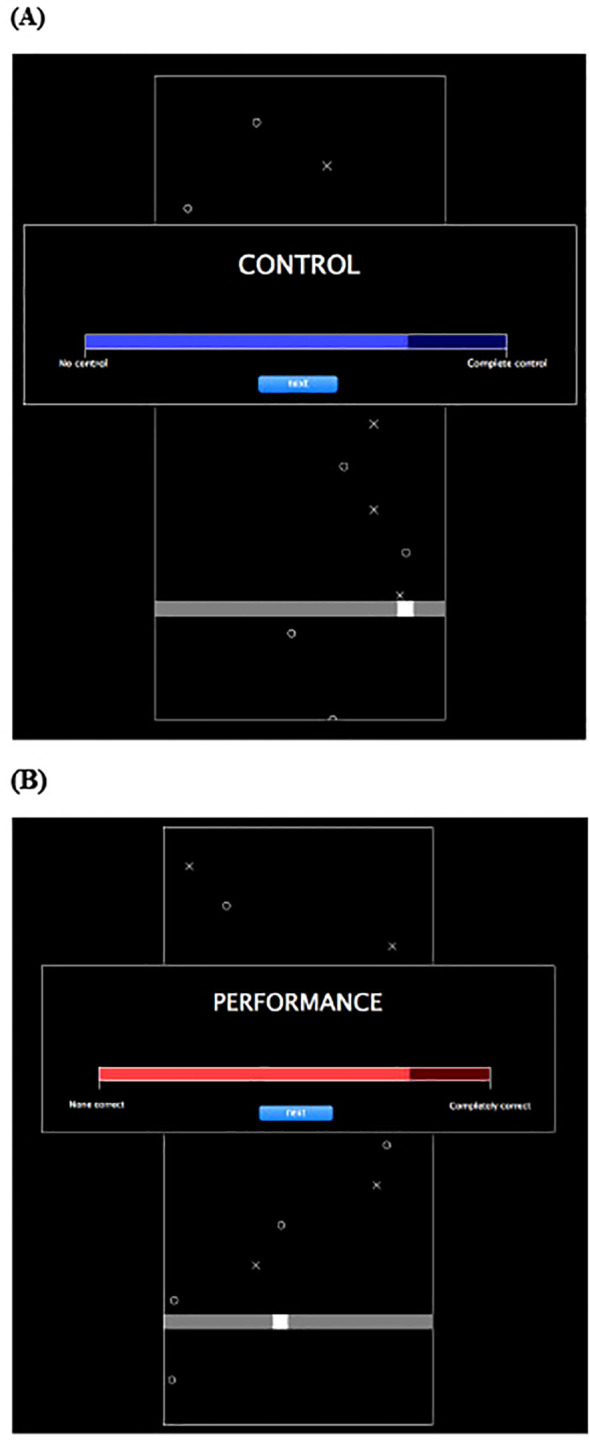
Magic and turbulence task.

The Magic and Turbulence Task will be used to measure participants’ sense of control (26). The Magic and Turbulence Task consists of four conditions (i.e., normal, lag, turbulence, magic) and two phases (i.e., game, judgment). Each phase manipulates participant’s control in the game. Specifically, the lag condition changes the cursor movement, the turbulence condition adds random movement to the position of the cursor, and the magic condition has the X’s disappear without the cursor touching the X. During the game phase, participants are asked to move a cursor along a horizontal track and catch downward falling X’s and miss falling O’s. During the judgment phase, participants are asked to rate their control over the game and performance during the game (**Figure 2**). Sense of control is assessed by calculating three summary control scores assessing participants’ awareness of their lack of control over and above their perception of their overt performance in conditions in which their control is manipulated (magic, turbulence and lag conditions). To calculate this, we will compute 3 summary control scores, namely, the contrast: (judgement of performanceC – judgement of controlC) – (judgement of performanceM– judgement of controlM) where the subscript C refers to the control or normal condition and M refers to the manipulated condition (magic, turbulence or lag). The Magic and Turbulence task is a well-validated measure of sense of control (26). The Magic and Turbulence Task is ecologically valid for FS because it reproduces the exact sensory-predictive disruptions that give rise to FS. As individuals recover, their capacity to detect, tolerate, and accurately interpret changes in control improves, and this task captures that improvement quantitatively.

#### 2.4.2 Catastrophic symptom expectations

##### 2.4.2.1 Salivary cortisol response to the cold pressor test

Salivary cortisol response to the CPT (ThermoFisher Scientific, USA) will be used as an objective biomarker to measure catastrophic symptom expectations. Previous research has established that cortisol response to the CPT corresponds to participants’ pain catastrophizing and perceived pain, and it is sensitive to within-subjects change, supporting its use as a measure of catastrophic symptom expectations in this study (27). The CPT uses an ARTIC A25 refrigerated bath with water maintained at 7 °C (± 1 °C). This temperature is 5.5°F warmer than a refrigerator and consistent with guidelines provided for the use of the CPT in children. It is a valid and reliable measure of catastrophic expectations in response to pain (27), and the CPT recreates the same stress-driven bodily sensations, catastrophic expectations, and autonomic responses that typically precede and maintain FS, allowing treatment-related improvements in these real-world mechanisms to be measured objectively. Participants will place their dominant hand in the refrigerated bath “for three minutes or until you can no longer tolerate the pain” and remove their hand from the bath after three minutes if not already removed. Blood pressure will be assessed prior to each CPT, and as noted, participants with uncontrolled hypertension (i.e., blood pressure >130/80 mmHg for adolescents ≥13 and systolic or diastolic blood pressure ≥95% based on sex and age for children younger than 13) will not be eligible to complete the task.

Salivary cortisol response to the CPT will be assessed using oral saliva samples obtained at 6 time points at each lab visit: 30 minutes pre-CPT, 15 minutes pre-CPT, five minutes pre-CPT, immediately post-CPT, 15 minutes post-CPT, and 30 minutes post-CPT. Participants will provide these samples by chewing on a Salivette (Sarsted, Leicester, UK) for 30–45 seconds or until completely saturated. Participants will be instructed to refrain from eating or drinking for at least 15 minutes prior and to not brush their teeth or eat foods that may cause gum bleeding for at least 2 hours prior to completing Salivettes. All lab visits assessing cortisol will be held between 2:00 PM and 5:00 PM given circadian variation in salivary cortisol levels (28). High sensitivity salivary cortisol immunoassay kits will be used to measure cortisol (Salimetrics, State College, PA, USA). After Salivettes are collected, the samples will be taken to the -80 degrees Celcius freezer for storage before a research assistant takes the sample to the appropriate personnel to be defroster, centrifuged, and analyzed.

##### 2.4.2.2 Pain catastrophizing questionnaire

The Pain Catastrophizing Scale (29) for Children (PSC-C) will also be used to measure catastrophic symptom expectations. Previous literature has demonstrated the measure as valid and reliable (29, 30).

##### 2.4.2.3 Pain tolerance

Pain tolerance to the CPT will also be used to assess catastrophic symptom expectations. Pain tolerance will be measured by the amount of time that patients keep their hand in the cool water. will be assessed by the amount of time patients keep their hand in the cool water.

### 2.5 Secondary outcome measures

Secondary outcomes will be assessed using questionnaires at multiple time points throughout the study. See **Table 1** for a summary of measures and when they will be administered. The questionnaires will assess FND and somatic symptoms, mood and quality of life, family dynamics and functioning, treatment engagement, beliefs, and alliance, illness cognitions and sense of control, and cognitive and sociodemographic background.

#### 2.5.1 Catastrophic symptom expectations

The Childhood Anxiety Sensitivity Index (CASI) (30) will be used to measure catastrophic symptom expectations and is valid and reliable. Immediately following the CPT at all lab visits, participants will rate their pain of a scale of 0 to 100 with 0 meaning “no pain” and 100 meaning “pain as intense as I can imagine.” Previous literature has demonstrated this measure to be valid in detecting treatment effects (31). Perceived pain severity to the CPT will be measured by the amount of time participants are able to keep their hand in the cool water during the CPT (32). Previous literature has demonstrated the CPT as a reliable, valid, and ethical way of assessing catastrophic symptom expectations in children (32, 33). To measure perceived pain severity, immediately following the CPT at all lab visits, participants will rate their pain of a scale of 0 to 100 with 0 meaning “no pain” and 100 meaning “pain as intense as I can imagine.” Previous literature has demonstrated this measure to be valid in detecting treatment effects (27).

#### 2.5.2 FND and somatic symptoms

Functional seizure diaries will be used to report FS frequency from 30 days before treatment begins until the 2-month follow-up. Parents and participants will provide these reports, and any discrepancies will be addressed by the therapist at each session to clarify frequency and improve accuracy. The Yale Global Tic Severity Scale (34) will be used to report tic frequency and severity for participants reporting tics. A self-report Other FND Symptoms Questionnaire will be used to assess frequency and severity of other FND symptoms (e.g., tremor, vision changing, paralysis).

The Children’s Somatic Symptom Inventory-24 will be used to measure somatic symptom severity. Internal reliability and validity were found to be adequate in a clinical sample with high test-retest reliability (35). The Level 2-Somatic Symptom self-reports will be used to measure somatic symptom severity. This self-report is adapted from the Patient Health Questionnaire Physical Symptoms (36).

#### 2.5.3 Mood and quality of life

The 16-item Anxiety Sensitivity Index (ASI (37); will be used to measure catastrophic symptom expectations. Internal consistency is adequate and test-retest reliability is high (38). The 47-item Revised Children’s Anxiety and Depression Scale (RCADS) will be used to measure symptoms of anxiety and depression. Internal consistency and test-retest reliability were good (39–41). The Dysfunctional Attitude Scale (DAS-9) will be used to measure the presence and intensity of dysfunctional attitudes that are associated with negative emotional states. Research on psychometric properties support the use of the measure and its subscales in the assessment of clinically depressed adolescents (42). The Columbia Suicide Severity Rating Scale (C-SSRS) will be used to assess suicide risk. Moderate to strong internal consistency suggest the tool is suitable for assessment of suicide risk in research settings (43).

The Quality of Life in Epilepsy for Adolescents (QOLIE-AD-48) is a 48-item questionnaire and will be used to assess health-related quality of life among participants. Reliability and validity were strong among adolescents with epilepsy (44), and it is used in patients with FS by instructing them to consider their FS when asked about seizures. The Functional Disability Index (FDI) will be used to measure the degree of physical and psychosocial functioning difficulties children experience due to their physical health. This questionnaire has high clinical utility and validity (45). The Rosenberg Self-Esteem scale and a single Quality of Life question will be used to measure self-esteem among participants. The Rosenberg Self-Esteem scale has high reliability, internal consistency, and convergent validity (46).

#### 2.5.4 Family dynamics and functioning

The Impact on Family Scale (IOFS) will be used to measure parent perception of the impact of their child’s FS on the family. Internal consistency is adequate and test-retest reliability is high (47). The Parenting Style and Dimensions Questions – Short Version (PSDQ-SV) will be used to assess the three main parenting styles: authoritative, authoritarian, and permissive. The PSDQ—Short Version has adequate validity across various cultural samples (48). The Beaver’s Self-Report Family Inventory will be used to measure parent and child perception of their family system’s functioning, mainly family competence and family style. The inventory has high internal consistency and test-retest reliability and good validity (49).

#### 2.5.5 Treatment engagement, beliefs, and alliance

The Treatment Preference Questionnaire will be used to assess which treatment the parent and participant prefer to be randomized to. The Credibility/Expectancy Questionnaire (CEQ) will be used to measure parent and participant treatment expectancy (i.e., beliefs regarding likelihood of improvement) and credibility (i.e., beliefs regarding whether the treatment appears logical and useful). Internal consistency is high and test-retest reliability is good for use in clinical studies (50). The Revised Helping Alliance Questionnaire will be used to measure the therapeutic alliance between the parent, patient, and therapist, and is valid across studies (51). The Clinical Global Impression Index will be used to rate the severity of the child’s illness. Psychometric properties of the CGI are good (52). The Child and Parent Weekly Questionnaires will be used to measure change in FS and other FND symptoms and use of the ReACT plan over the previous week. The Child and Parent Follow-Up Forms will be used to assess patient and parent beliefs regarding the efficacy of ReACT and cause of their FS.

#### 2.5.6 Illness cognitions and sense of control

The Mishel Uncertainty in Illness Questionnaire (MUIS) will be used to measure the patient’s level of uncertainty regarding their FS and course of treatment. Research among adolescents suggests strong research utility (53). The Illness Cognitions Scale (ICS) will be used to assess the patient’s difficulty adjusting out of the sick role. Subscales will include Helplessness, Acceptance, and Perceived Benefits. Prior literature demonstrates adequate internal consistency and reliability among adolescents (54).

A single Sense of Agency question will be used to assess participant’s sense of control over their FS (i.e., “How much control do you believe you have over your episodes?”). Responses will include “complete control”, “a lot of control, but not complete control”, “a little control”, and “no control”. The Multidimensional Health Locus of Control Scales (MHLCS) will be used to assess participant beliefs regarding health-related behaviors. Specifically, Form A (MHLCS – Form A) will be used to assess the extent to which the participant believes their own behaviors are the source of reinforcement for health behaviors (i.e., internal locus of control; “If I get sick, it is my own behavior which determines how soon I get well again”) and Form C (MHLCS – Form C) will be used to assess the extent to which the participant believes the behaviors of others are the source of reinforcement for health behaviors (i.e., external/’powerful others’ locus of control; “Having regular contact with my physician is the best way for me to avoid illness”). Validity and reliability were good (55, 56).

#### 2.5.7 Cognitive and sociodemographic background

A demographics questionnaire will be used to assess sex, gender, sexuality, preferred pronouns, height, and weight. The Parent and Child Mini-Forms will be used to assess data regarding FS (e.g., data of FS onset and diagnosis. presence of premonitory symptoms) at baseline. The Parent and Child Mini-Forms will also be used to assess other baseline characteristics (e.g., school status, medication use, hospital visits), and this data will be collected again via the Child and Parent Follow-Up Forms. The COVID-19 FND Questionnaire will be used to assess participants’ COVID-19 history.

The Shipley Institute of Living Scale (Shipley IQ) will be used to assess crystallized and fluid cognitive abilities. The Shipley IQ has demonstrated good reliability and validity across clinical and non-clinical samples (57). The Healthcare-Related Stigma Questionnaire will be used to assess FS-related experiences with healthcare providers. The Childhood Trauma Questionnaire (CTQ) will be used to assess history of physical, sexual, and emotional abuse, and physical and emotional neglect. Prior literature demonstrates good test-retest reliability and internal consistency across a range of samples (58).

### 2.6 Interventions

The first session for both the ReACT and supportive therapy intervention arms will be conducted in-person at UAB and last 1.5 hours. This visit will be followed by 11 one-hour telehealth sessions, resulting in an overall equivalent intervention dose across intervention arms. The first therapy session will be held approximately one week after the baseline visit. Subsequent sessions will typically be scheduled weekly such that the participant will complete the intervention in 3 months. However, should therapy sessions not occur weekly due to illness, holidays, vacation, etc., the duration of time between the first and final sessions will be limited to five months.

Participants will be offered the treatment to which they were not randomized after the 2-month follow-up when the study is completed. Study investigators and staff will not provide any medication-related advice to participants. Both supportive therapy and ReACT interventionists will be master’s level therapists. All interventionists will receive extensive training by the PI and last author (AF) on their respective intervention, participate in weekly supervision, and complete yearly training sessions.

#### 2.6.1 Retraining and control therapy

The protocol for ReACT has been described in previous publications (4, 59). In short, for participants randomized to ReACT, the first session will include: 1) an etiological explanation of FND, 2) an individualized plan for retraining FND symptoms, 3) a family and friend plan for responding to FND symptoms, and 4) a plan to return to school and activities. Therapists will conduct this and all subsequent sessions using the ReACT Precision Treatment Tool, an adaptive digital manual that tailors session content to the issues most relevant to each patient at that time. Guided by this tool, subsequent sessions will focus on modifying and adapting the patient’s individualized plan based on patient progress and needs, addressing symptom triggers and avoidance behaviors, generalizing CBT skills to manage stressors or mood concerns, and developing a relapse prevention plan. Each session will include both individual and joint discussions with the participant and parent/guardian.

#### 2.6.2 Supportive therapy

Supportive therapy is an unstructured therapy based on the assumption that relief from problems can be achieved by discussion with a therapist (60). The purpose of supportive therapy is not to acquire new skills or find solutions to problems. The etiological model provided to patients will focus on FS as the result of stress or mood. Supportive therapy sessions will focus on discussion of stressors or mood concerns and how they are related to their FS, and therapists will provide empathy. Patients will be asked about things that help them deal with stressors, but consistent with supportive therapy protocols, specific strategies will not be generated by the therapist (60). Each session will be the same duration as ReACT sessions and follow the same structure as the ReACT intervention (i.e., individual and joint discussions with the participant and parent/guardian) to further ensure dose equivalence with ReACT.

To minimize expectancy bias, both ReACT and supportive therapy will be described to families as credible interventions that may help manage FS and improve coping. Participant-related treatment expectancy will be assessed at the first therapy session using the Credibility/Expectancy Questionnaire (described in section 2.5.5).

#### 2.6.3 Treatment fidelity

All ReACT and supportive therapy sessions will be videotaped, and trained research assistants will review and score 20% of the sessions using standards of published guidelines (61, 62) to ensure treatment fidelity and no contamination between behavioral interventions. Separate fidelity checklists for ReACT and supportive therapy will be used to ensure each treatment is delivered as intended, with items rated for both presence and quality. Fidelity will be calculated as the percentage of total possible points earned, based on the sum of item scores divided by the checklist’s total point value. For each therapist’s first 5 participants, two research assistants will score the same sessions until the scoring is above 95% agreement to ensure validity of fidelity ratings. Adequate fidelity will be defined as scoring above 80% threshold. If fidelity is insufficient for any of the therapists’ first patients, additional training will be provided until fidelity is reached. Fidelity will be re-assessed at 3- and 6-months after enrollment begins and before final analyses.

#### 2.6.4 Participant retention

All efforts will be taken to ensure enrolled and randomized participants are followed for the entire study period. To facilitate this, participants will be asked to provide their contact number and email address, their parent/guardian’s contact number and email address, and an additional contact number who can be reached out to in the event the participant or parent/guardian cannot be reached. Further, reminder calls will be made the day before all appointments and follow-up visits will be scheduled over Zoom prior to therapy appointments. Follow-up appointments will also be offered at flexible times with weekend and before and after school availability.

Using an intent-to-treat analysis, participants who choose to withdraw from the treatment but wish to complete remaining study visits will complete a lab visit 7 days after withdrawing and 2-months later. Participants who do not ask to withdraw from the study or treatment, but no-show their therapy session or lab visit, will be called the day of their appointment. If they cannot be reached, a voicemail will be left including call back information. If the participant does not respond within three days, another call will be made. If after one week there has been no communication from the participant, a final call will be made to the participant, parent/guardian, and additional contact. The participant will be considered lost to follow-up if no response is provided after this point.

### 2.7 Data analysis plan

Post-treatment sense of control and catastrophic symptom expectations will be analyzed using separate ANCOVA models adjusting for pre-treatment values. The primary analysis will compare these outcomes between the ReACT and supportive therapy groups.

The relationship between changes in sense of control, catastrophic symptom expectations, and FS frequency will be assessed by examining the total association of each target with FS and potential interactions. Since FS frequency is a count variable, negative binomial regression will be used for each target, adjusting for baseline FS frequency and baseline sense of control. Interactions between cortisol response to the CPT and the three magic and turbulence task conditions will also be explored.

Negative binomial regression will also be used to confirm the preliminary efficacy of ReACT in reducing FS frequency and determine the feasibility of a fully powered clinical trial. Post-treatment FS frequency will be modeled using treatment group assignment, baseline FS frequency, and relevant covariates. Changes in FS frequency from one-week and 2-month follow-ups will be analyzed using adjusted linear models to account for baseline measures and potential confounding factors. Change in functional outcomes (e.g., school attendance, quality of life, family functioning) will also be assessed.

All statistical analyses will be conducted using SPSS. Sensitivity analyses will assess the robustness of findings. Missing data will be handled using multiple imputation methods where appropriate, and statistical significance will be set at p < 0.05 for primary hypotheses with adjustments for multiple comparisons made as needed. Model assumptions will be checked to ensure the validity of results, and alternative modeling approaches will be considered if deviations from standard assumptions are detected.

#### 2.7.1 Power and sample size

A Cohen’s d estimate of effect size of 0.5 was determined based on effect sizes from previously published studies using the Magic and Turbulence Task (26, 63, 64) and assessing change in pain sensitivity, pain tolerance, and cortisol for the CPT (33, 65, 66). A power analysis determined a sample size of N = 64 will be required to obtain >80% power at the two-tailed 0.05 alpha level to detect differences in the ReACT and supportive therapy interventions. However, 80 participants will be recruited to account for a 20% dropout rate.

## 3 Discussion

Given the limited success of targeting mood and trauma-related factors in reducing FS symptoms, the present study represents a critical step toward identifying treatment targets which impact FS frequency for pediatric patients with FS. Establishing catastrophic symptom expectations and sense of control as treatment targets for ReACT is critical for advancing treatment for pediatric FS. Further, investigating the relationship between changes in these targets and FS frequency may provide insight into the mechanisms underlying the onset of FS as well as symptom persistence and remission, thus informing further individualized interventions.

The integration of the ReACT Precision Treatment Tool as a provider-centered platform for tracking patient progress and optimizing intervention delivery is also promising. This tool has the potential to enhance treatment fidelity and dissemination, thereby increasing the accessibility of ReACT in clinical settings. Additionally, by assessing the effects of treatment over a two-month period, this study will generate data on the sustained effects of ReACT compared to supportive therapy. If sustained improvements are observed, future research may explore strategies to maintain and enhance these gains.

Finally, the findings from this study will inform the feasibility of a larger-scale R01 trial further exploring the mechanisms by which ReACT effective in reducing FS and confirming the impact of ReACT in a fully-powered efficacy trial. Ultimately, this research has the potential to help elucidate the treatment targets that can optimize treatment outcomes for adolescents and children and establish a gold-standard treatment for pediatric FS, improving patient outcomes and reducing the burden on families and healthcare systems.

## 4 Strengths, limitations, and additional considerations

The present study has several notable strengths, including a focus on identifying mechanistic treatment targets for pediatric FS beyond mood and trauma-related factors, with direct relevance to FS frequency and clinical outcomes. Further, the integration of objective biomarkers as measures of treatment response and provider-centered tools to support fidelity and dissemination represent an important step toward optimizing and scaling evidence-based interventions for pediatric FS. At the same time, several limitations and broader considerations warrant note. First, although the Magic and Turbulence Task has been shown to be sensitive to change in pediatric FS and other conditions, formal evaluation of test-retest reliability and practice effects has not yet been established.

Second, although the CPT is a well-established measure for assessing pain catastrophizing and perceived pain, the present study does not capture responses to other physiological sensations (e.g., increased heart rate) or cognitive-affective responses (e.g., catastrophizing) in everyday contexts. Future studies will benefit from incorporating multiple complementary tasks to more comprehensively assess catastrophic symptom expectations across domains. Finally, although the present design intentionally focused on pediatric FND, the biobehavioral processes examined may have implications for understanding and informing intervention approaches in adult FND, representing an important avenue for future translational research.

## 5 Ethics and dissemination

All study staff will receive extensive training in ethical principles and procedures and all procedures will be monitored by the PI and senior staff. Results will be disseminated via conference presentations and published in peer reviewed journals. See below additional ethical considerations.

### 5.1 Data safety monitoring board

The Data Safety Monitoring Bodard (DSMB) will oversee key aspects of the study and provide recommendations to the Principal Investigator, and the funding agency. This includes monitoring enrollment and randomization, such as the number of patients approached, consented, and retained in both the treatment and control groups, assessing any adverse events related to the study protocol, reviewing the demographic characteristics of participants, and ensuring the ethical collection of data and responsible data analysis. DSMB members will have no financial or other conflicts of interest with any collaborating or competing organizations involved in the study. The PI will take ultimate responsibility for data safety monitoring and reporting in the study.

### 5.2 Confidentiality and security

All data collected for this study will be securely stored in REDCap (67, 68)—an online software platform—behind the UAB firewall in accordance with Institutional Review Board and Health System Information Services requirements for data protection. Data entered into REDCap are password protected and only accessible to designated study staff. Designated study staff will be trained on data security procedures and confidentiality, and access logs will be reviewed quarterly to ensure compliance. To further ensure confidentiality and security, enrolled participants will be assigned an individual study ID number, and only their date of birth will be stored with this ID number in REDCap. All data will be identified only by this numeric code. While a master list linking names, contact numbers, and ID numbers will also be stored to facilitate the collection of follow-up data, this password protected spreadsheet will be stored in a separate location and only accessible to the senior staff. All other assessment data (e.g., computer task data) will be generated by computer task programs and merged into a database or hand-entered into a computer and merged into a database. Hand-entered forms and other related documents will be stored in a secure location in a locked filing cabinet. Following study completion, data will be exported from REDCap into an Excel document, then uploaded to SPSS for analysis.
